# Adaptive cluster algorithm to infer Boltzmann machines from multi-electrode recording data

**DOI:** 10.1186/1471-2202-12-S1-P224

**Published:** 2011-07-18

**Authors:** Simona Cocco, Rémi Monasson

**Affiliations:** 1 CNRS-Laboratoire Physique Statistique de l'ENS, Paris, 75005 France; 2 The Simons Center for Systems Biology, Institute for Advanced Study, Einstein Drive, Princeton NJ 08540,USA

## 

Multi-electrode recordings allow the recording of the activity of a neural population of tens to hundred cells over periods of hours. Two examples are given by the recording of the activity of ganglion cells in the retina [1-3,6] , and the recording of the activity of the prefrontal cortex in behaving rats [4,5]. Two important issues in neuroscience are 1) to find a predictive model able to reproduce the statistical features of the recorded activity, as the spiking frequencies of the cells, the two-cell correlations and the occurrence of multi-cell patterns; 2) to infer from the recorded activity some functional couplings between cells, which could give an insight about neural circuits. Schneidman et al. [2] and Shlens et al. [3] have first used the Ising model to analyze retina recordings, as a Boltzmann machine (BM) able to reproduce both the average activity of the cells and the pairwise correlations between cells in an equal time window of size Dt. However efficient algorithms to infer the Ising couplings for a large population of cells remain to be found. In addition, an important question is how to deal with time (finite recording time) and space (small area recorded) undersampling.

In the present work we propose a new and efficient algorithm to infer fields and pairwise couplings of an Ising model from the data. Our procedure considerably improves over the algorithm presented in [7] and is based on an adaptive cluster expansion of the cross entropy between the Ising model and the data. The interaction network is progressively unveiled, through a recursive processing of larger and larger subsets of variables, which we call clusters. To each cluster is associated an entropy contribution which assesses how much the cluster is relevant to infer the BM. Clusters such that the entropy contribution is smaller than a fixed threshold are discarded; the other clusters are kept and recursively used to generate larger clusters. The threshold must be large enough to avoid overfitting of the data corrupted by the sampling noise and small enough in order not to miss important components of the interaction network. Contrary to previous cluster expansions [7], the number, size, and composition of the clusters automatically adapt to the data, and, rather than the sole size of the cells population determine the running time of the algorithm. We provide a pseudo-code for the practical implementation of our algorithm and intend to release soon an open-access code.

Our procedure has been validated on synthetic data sets, and used to re-analyze multi-electrode recordings of neural cells of the activity of salamander ganglion cells previously published in [2,6] and of the activity in a region of the prefrontal cortex of a behaving rat previously published in [4,5] . The algorithm can efficiently deal with population size that varied on the data sets from 32 cells to 120 cells. To illustrate the potential applications we have tested the dependence of couplings upon stimulus on two data sets registered by Schnitzer and Meister [6] from the same retina in dark condition (spontaneous activity) and with a random flickering checkerboard. We have compared for each pair of cells i,j, the values of the interactions J_ij_ inferred from Dark and Flicker. We have found that most of the couplings are conserved under the two stimuli but some pairs of neurons with large interactions in Flicker have weak couplings in Dark. We have used the inferred couplings to draw retinal maps in the receptive fields plane of the cells. For Dark, the largest coupling map define a planar graph with short range (almost nearest neighbor) connections. For flicker the strong non conserved couplings pointed out in the previous paragraph often are long-range interactions.

We will discuss some important aspects of the Ising model such as: How do couplings change with the removal of cells from the recording? What temporal correlations are neglected in the Ising model (dependence on the bin size Dt)? How do couplings inferred with a dynamical model (Integrate and Fire) compare with Ising couplings (see Abstract by Carlo Barbieri, Simona Cocco, Remi Monasson submitted to the present conference)?
